# Investigating the Interaction of Cyclic RGD Peptidomimetics with α_V_β_6_ Integrin by Biochemical and Molecular Docking Studies

**DOI:** 10.3390/cancers9100128

**Published:** 2017-09-21

**Authors:** Monica Civera, Daniela Arosio, Francesca Bonato, Leonardo Manzoni, Luca Pignataro, Simone Zanella, Cesare Gennari, Umberto Piarulli, Laura Belvisi

**Affiliations:** 1 Dipartimento di Chimica, Università degli Studi di Milano, via Golgi 19, I-20133 Milano, Italy; monica.civera@unimi.it (M.C.); francesca.bonato@studenti.unimi.it (F.B.); luca.pignataro@unimi.it (L.P.); simone.zanella@unimi.it (S.Z.); cesare.gennari@unimi.it (C.G.); 2 Istituto di Scienze e Tecnologie Molecolari (I.S.T.M.), Consiglio Nazionale delle Ricerche (C.N.R.), via Golgi 19, I-20133 Milano, Italy; daniela.arosio@istm.cnr.it (D.A.); leonardo.manzoni@istm.cnr.it (L.M.); 3 Dipartimento di Scienza e Alta Tecnologia, Università degli Studi dell’Insubria, via Valleggio 11, I-22100 Como, Italy; umberto.piarulli@uninsubria.it

**Keywords:** RGD peptidomimetics, integrins, molecular docking, binding assays

## Abstract

The interaction of a small library of cyclic RGD (Arg-Gly-Asp) peptidomimetics with α_V_β_6_ integrin has been investigated by means of competitive solid phase binding assays to the isolated receptor and docking calculations in the crystal structure of the α_V_β_6_ binding site. To this aim, a rigid receptor-flexible ligand docking protocol has been set up and then applied to predict the binding mode of the cyclic RGD peptidomimetics to α_V_β_6_ integrin. Although the RGD interaction with α_V_β_6_ recapitulates the RGD binding mode observed in α_V_β_3_, differences between the integrin binding pockets can strongly affect the ligand binding ability. In general, the peptidomimetics exhibited IC_50_ values for integrin α_V_β_6_ (i.e., the concentration of compound required for 50% inhibition of biotinylated fibronectin binding to isolated α_V_β_6_ integrin) in the nanomolar range (77–345 nM), about 10–100 times higher than those for the related α_V_β_3_ receptor, with a single notable ligand displaying a low nanomolar IC_50_ value (2.3 nM). Insights from the properties of the binding pocket combined with the analysis of the docking poses provided a rationale for ligand recognition and selectivity.

## 1. Introduction

Integrins are αβ-heterodimeric transmembrane proteins that are involved in cell adhesion and signaling [1,2]. Because of their central role in a variety of physiological cell functions, as well as in the pathobiology of many diseases, integrins continue to attract interest for the development of therapeutic agents [3,4,5,6,7]. For instance, in cancer, pharmacological research is focused on a group of integrins that play key roles in tumor angiogenesis, progression, and metastasis, and share the property to recognize ligands containing the RGD (Arg-Gly-Asp) sequence, with a specificity determined by the features of the binding pocket and the context of the ligand RGD motif (i.e., flanking residues, conformation) [8,9,10].

Driven by the functional roles and the upregulated expression on various tumor cells of several subtypes from the RGD-binding subfamily, such as α_5_β_1_ along with α_V_β_3_ and other α_V_ integrins, extensive chemical research has been carried out to develop RGD-based peptidic and peptidomimetic integrin ligands as inhibitors of integrin functions and as targeting devices for the selective delivery of drugs or imaging probes to tumors [9,10,11,12,13,14,15,16].

**Figure 1:**
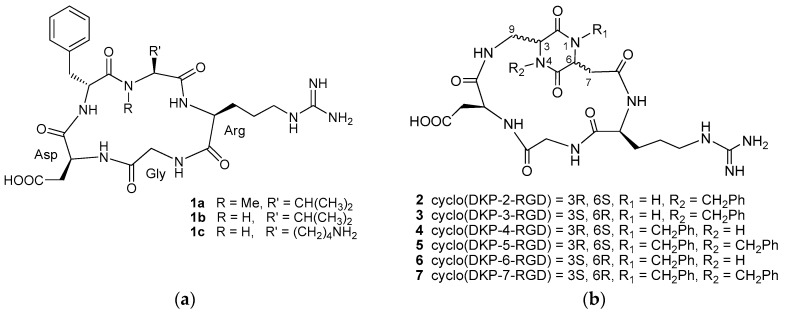
(**a**) Cyclic RGD (Arg-Gly-Asp) pentapeptides **1a**–**c**; (**b**) Cyclic RGD peptidomimetics **2**–**7** containing DKP scaffolds.

Among them, the best known RGD peptide is the cyclic pentapeptide cyclo[RGDf(N-Me)V] **1a** (Cilengitide, Figure 1a) [17], the first integrin antagonist to be tested in clinical trials [18], that is currently undergoing phase II studies for the treatment of several cancer types after its failure in a phase III trial for the treatment of patients with newly diagnosed glioblastoma [19,20]. Other well-known RGD peptides are cyclo[RGDfV] **1b**, the parent peptide of Cilengitide, and cyclo[RGDfK] **1c**, that has been extensively used as targeting motif for targeted cancer diagnosis and therapy studies (Figure 1a) [10,13,14,15,16,21].

With the aim of developing new small molecule integrin antagonists with improved properties, we have recently synthesized a small library of cyclic RGD peptidomimetic integrin ligands, containing bifunctional diketopiperazine (DKP) scaffolds that differ in the configuration at the two DKP stereocenters and in the substitution at the DKP nitrogen atoms, and that can be viewed as conformationally constrained dipeptide mimics formed by two β-amino acids (Figure 1b) [22,23,24].

In particular, the cyclic RGD peptidomimetics **2**–**7** derived from *trans*-DKP scaffolds (DKP2-DKP7) were shown to bind α_V_β_3_, α_V_β_5_, and α_5_β_1_ integrins with a preferential affinity towards α_V_β_3_, inhibiting the binding of biotinylated vitronectin to the purified α_V_β_3_ integrin at low- or sub-nanomolar IC_50_ values [24,25]. The interaction of the cyclic DKP-RGD peptidomimetics with α_V_β_3_ and α_5_β_1_ integrins has been investigated by means of integrated spectroscopic and computational studies, gaining insights into the molecular basis of their activity [24,25,26]. In particular, the preferred ligand conformations, displaying an extended arrangement of the RGD motif with a distance of about 9 Å between the Cβ atoms of Asp and Arg, are highly preorganized for the interaction with integrins α_V_β_3_ and α_5_β_1_, as demonstrated by the docking studies in the crystal structures and NMR experiments with α_V_β_3_-rich bladder cancer cells and α_5_β_1_-rich breast cancer cells [24,25,26].

Recently, ligand **3** (Figure 1b) was reported to display inhibitory effects on the FAK/Akt integrin-activated transduction signaling pathway and on integrin-mediated cell infiltration processes in human glioblastoma cells, thus qualifying as a true integrin antagonist [27]. It was also shown to significantly inhibit the cell adhesion of different cancer cells, and angiogenesis induced by pro-angiogenic growth factors in human endothelial cells [28]. Moreover, after suitable functionalization, it was exploited as a targeting agent for the preparation of conjugates designed to release cytotoxic drugs selectively within cancer cells expressing α_V_β_3_ integrin [29,30,31].

In this context, information on the ability of small RGD molecules to interact with closely related integrin subtypes involved in cancer, is crucial to fully understand the biological activity profiles and to develop suitable integrin ligands for the modulation of integrin functions or the targeted delivery of chemotherapy [9,10,13,14,15,16,21].

Prompted by its pathological relevance in cancer and by the availability of X-ray structural information [32], we became interested in the integrin α_V_β_6_, one of five α_V_ integrins and the unique β_6_ integrin from the RGD-binding subfamily. α_V_β_6_ is expressed on epithelial cells, especially during development, after injury or inflammation, or on many carcinomas. The ligand binding site of α_V_β_6_ is in the N-terminal head region formed by the interaction of the α_V_ β-propeller domain with the β_6_ βI domain. α_V_β_6_ preferentially binds to the latency-associated peptide (LAP) of the transforming growth factor-β (TGF-β), but can also recognize the matrix proteins fibronectin and tenascin. In this regard, α_V_β_6_ interacts with the LAP/TGF-β complex by binding with the RGD motif present in the LAP peptide, thus breaking the latency complex and releasing the active form of TGF-β which, in turn, binds to and activates its receptors on the cell surface [32,33]. In particular, similarly to integrin α_V_β_8_, α_V_β_6_ is specialized to activate TGF-β1 and TGF-β3 from large latent complexes. Therefore, high α_V_β_6_ expression in carcinomas may contribute to progression through its effects on TGF-β activity.

In the framework of a study investigating the determinants of α_V_β_6_ high specificity for the RGD motif present in the prodomain of TGF-β1 and TGF-β3, the crystal structures of the α_V_β_6_ headpiece with or without a pro-TGF-β3 undecapeptide have been recently solved [32]. The ligand-bound structure revealed that α_V_β_6_ recognizes not only RGD but also the adjacent C-terminal LGRLK motif that folds into an amphipathic α-helix fitting into a hydrophobic pocket in the β_6_ subunit.

Herein, we report on the interaction of our cyclic DKP-RGD peptidomimetics with α_V_β_6_ integrin by means of competitive solid phase binding assays to the isolated receptor and docking calculations in the crystal structure of the α_V_β_6_ binding site. Starting from the structure of ligand-bound α_V_β_6_, a rigid receptor-flexible ligand docking protocol has been set up and then applied to predict the binding mode of the cyclic RGD peptidomimetics to α_V_β_6_ integrin. The analysis of the properties of the receptor pocket, combined with the examination of the docking poses allowed to rationalize the experimental binding affinities for the α_V_β_6_ integrin, which turned out to be about 10–100 times lower than those for the related α_V_β_3_ receptor. On the basis of docking calculations, the best cyclic RGD peptidomimetic was also identified, displaying a low nanomolar IC_50_ value.

## 2. Results

### 2.1. Integrin Receptor Competitive Binding Assays

The cyclic peptidic (**1a**–**c**) and peptidomimetic (**2**–**7**) RGD ligands were examined in vitro for their ability to compete with biotinylated fibronectin for binding to the isolated α_V_β_6_ integrin (Table 1). The assay was performed according to previously reported procedures [24,25] with slight modifications. In particular, a concentration of 1 μg/mL of integrin receptor was required for obtaining an efficient coating of the plates and a good reproducibility of the data [21,34]. Various concentrations (10^−11^–10^−4^ M) of the RGD ligands in the presence of biotinylated fibronectin (1 μg/mL) were then added to the plates and finally bound fibronectin was revealed by using a streptavidin-biotinylated peroxidase complex (see Section 4 for a detailed description).

To validate the binding assay protocol, the well-known cyclopeptidic integrin ligands **1a**–**c** were first assayed. Both compounds **1b** (c[RGDfV]) and **1c** (c[RGDfK]) showed binding affinities for integrin α_V_β_6_ (expressed as the ligand concentration required for 50% inhibition of endogenous ligand binding) comparable with data recently reported in literature [21] (Table 1). A nanomolar IC_50_ value similar to that of the other cyclopeptides was observed also for **1a** (c[RGDf(N-Me)V], IC_50_ = 82.8 ± 4.9 nM), in contrast with the micromolar value recently reported [21]. It must be noted that the two IC_50_ values are obtained by competitive solid phase binding assays by using two different procedures and two different α_V_β_6_ natural ligands: immobilized integrin and soluble fibronectin in the present assay, immobilized latency associated peptide (LAP), and soluble integrin in the other [21]. Noteworthy, the present result is compatible with the outcomes of docking calculations (vide infra).

All of the cyclic DKP-RGD ligands **2**–**7** showed binding affinities for integrin α_V_β_6_ lower than those for α_V_β_3_, displaying a selectivity ratio (IC_50_ α_V_β_6_/IC_50_ α_V_β_3_) ranging from about 10 (**4**, **5**, **7**) to nearly 100 (**2**) (Table 1). The trend is confirmed by the reference cyclopeptides, exhibiting IC_50_ values for α_V_β_6_ about 35 (**1b**, **1c**)–100 (**1a**) times higher than those for α_V_β_3_. Among the RGD peptidomimetics, compound **7** proved to be the best α_V_β_6_ ligand, inhibiting the binding of biotinylated fibronectin to α_V_β_6_ at a low nanomolar IC_50_ value. Interestingly, this ligand was also the most potent α_V_β_3_ ligand of the series, displaying a subnanomolar IC_50_ value.

As a negative control in the determination of binding activities, a cyclic peptidomimetic containing the Arg-Ala-Asp (RAD) sequence was prepared and tested (compound **8**, c[DKP-3-RAD], see Supplementary Materials, Scheme S1 and Figures S1–S6), displaying micromolar IC_50_ values with both α_V_β_3_ and α_V_β_6_ integrins (Table 1).

### 2.2. Docking Model of α_V_β_6_ Integrin and X-ray Structure Analysis

The computational model for the interaction of RGD ligands with the α_V_β_6_ integrin was developed by means of docking calculations using Glide V5.7 [35] (see Section 4 for a detailed description), starting from the X-ray structure of the extracellular segment of integrin α_V_β_6_ in complex with the RGD-containing undecapeptide of the TGF-β3 prodomain (PDB code: 4UM9) [32].

In the crystal structure, the headpiece of α_V_β_6_ adopts a closed conformation [32,36] similar to that adopted by α_V_β_3_ in the X-ray complex with Cilengitide [37]. In both of the crystal complexes the RGD sequence shows an extended conformation characterized by a Cβ(Arg)-Cβ(Asp) distance of 8.9 (α_V_β_3_)–9.4 (α_V_β_6_) Å, and a separation between the charged Arg and Asp side chains of 13.7 (α_V_β_3_)–14.2 Å (α_V_β_6_) (measured between the carbon atoms of the guanidinium and carboxylate groups). As observed in other X-ray structures of integrins in complex with RGD ligands [37,38,39,40], the RGD sequence binds at the interface of the α and β subunits with the carboxylic and guanidine groups acting as an electrostatic clamp, respectively, on a bivalent cation of the β subunit (MIDAS, metal ion-dependent adhesion site) and on specific acid residues of the α subunit. The oxygen atom of RGD aspartate side chain not engaged by MIDAS Mg^2+^ ion forms hydrogen bonds with backbone NH groups of β_6_-Asn218 and β_6_-Ala126. The arginine of RGD makes a bidentate side-on interaction through the guanidinium group to the side chain of Asp218 in the α_V_ subunit, as in binding to α_V_β_3_, but does not interact with α_V_-Asp150 side chain as for Cilengitide in α_V_β_3_. Other stabilizing hydrogen bond interactions occur between the backbone Gly carbonyl and Asp-NH ligand moieties and β_6_-Thr221 side chain and β_6_-Ile219 carbonyl group, respectively. It is worth noting that the salt bridge between α_V_-Asp219 and β_3_-Lys253 at the RGD binding site interface cannot be formed in α_V_β_6_ due to β_6_-Asp256 mutation, making the α_V_-Asp219 residue more accessible to the interaction with the ligands.

Large differences in the ligand-binding region between α_V_β_6_ and α_V_β_3_ are also represented by point mutations in the β2-β3 loop and in two neighboring interacting loops [32]. The three residues forming π-cation interactions in β_3_, Tyr166, Arg214, and Arg216, are replaced in β_6_ by Lys170, Ala217, and Ile219. Furthermore, β_3_-Tyr122 residue, which is engaged into a π-interaction with the Phe residue of Cilengitide, is mutated into β_6_-Ala126. In addition to RGD interactions, the immediately following LGRLK sequence of the TGF-β3 peptide forms an amphipatic α-helix that extensively interfaces with the β_6_ subunit exploiting a hydrophobic pocket close to the RGD binding site. Most of the contacts are formed with Ala126, Ser127, Cys180, Ile183, Tyr185, Ala217 side chains, and Pro179 backbone atoms.

**Figure 2:**
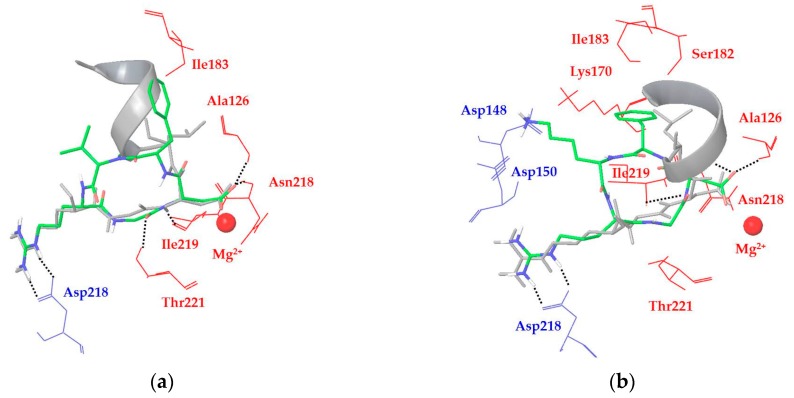
Docking best poses of (**a**) ligand **1a** (green) and (**b**) ligand **1c** (green) overlaid to the X-ray structure of the TGF-β3 undecapeptide (grey, α-helix represented as a ribbon) into integrin α_V_β_6_ (from 4UM9.pdb). Only selected integrin residues involved in interactions with the ligand are shown and labeled in blue for α_V_ and red for β_6_. Non-polar hydrogens are hidden for clarity, while intermolecular hydrogen bonds are shown as black dashed lines.

In all of the docking calculations, the X-ray binding mode of the RGD motif with the α_V_β_6_ integrin was taken as a reference model for the analysis of the docking results in terms of ligand–protein interactions. For instance, ligands **1a** and **1c** are able to reproduce the experimentally determined binding mode of the RGD sequence (Figure 2), even if some hydrogen bond interactions are not optimal in all the calculated docking poses. More importantly, **1a** and **1c** cannot significantly improve the interaction with the α_V_β_6_ binding site as gained by their D-Phe residue in the α_V_β_3_ pocket (due to β_3_-Tyr122 mutation into β_6_-Ala126) or by the TGF-β3 α-helix in the β_6_-specific hydrophobic pocket. These considerations might explain the reduced binding affinities of these cyclic peptides for integrin α_V_β_6_ when compared to α_V_β_3_. In particular, the docking poses of ligand **1a** (Cilengitide) show the D-Phe aromatic group in contact with β_6_-Ile183 and β_6_-Ala126, fitting only partially the α-helix region and the corresponding hydrophobic pocket (Figure 2a). Conversely, the docking poses of ligand **1c** display electrostatic interactions between the ligand Lys side chain and the integrin α_V_-Asp150 and α_V_-Asp148 residues, forcing the ligand D-Phe aromatic moiety in proximity of β_6_-Lys170, β_6_-Ser182, and β_6_-Asn218 residues, far away from the α-helix binding site (Figure 2b).

### 2.3. Docking of Cyclic DKP-RGD Peptidomimetics into α_V_β_6_ Integrin

**Figure A1:**
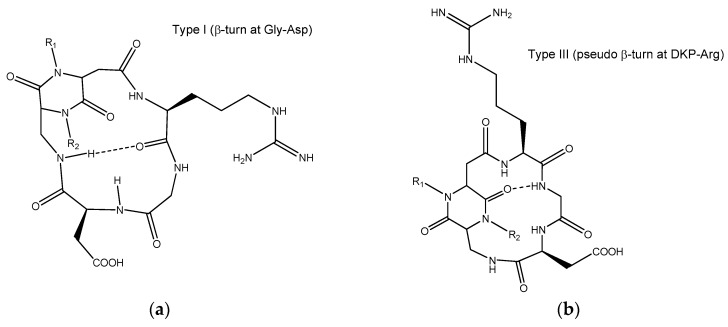
(**a**) 2D representation of type I geometry; (**b**) 2D representation of type III geometry.

Docking studies were performed starting from the macrocycle conformations of the cyclic DKP-RGD peptidomimetics that have been previously reported [24] and are shown in Appendix A (Figure A1) and in the Supplementary Materials (Figures S7–S9). When compared to the X-ray RGD bound conformation, both type I (characterized by a distorted β-turn at Gly-Asp) and type III (characterized by a pseudo-β-turn at DKP-Arg) geometries preferentially adopted by these ligands, display a similar extended arrangement of the RGD sequence satisfying the pharmocophoric requirements for the binding to integrin α_V_β_6_. Accordingly, docking results show that the top-ranked binding modes of all of the ligands maintain the key interactions observed for the RGD motif into the X-ray complex. The Asp and Arg side chains fit into the corresponding charged regions of the receptor, coordinating the MIDAS ion and forming the bidentate side-on salt bridge with α_V_-Asp218, respectively. The hydrogen bonds between the oxygen atom of Asp side chain and backbone NH groups of β_6_-Asn218 and β_6_-Ala126 are also present, as well as further stabilizing H-bonds involving ligand Gly and Asp backbone moieties and β_6_-Ile219 and β_6_-Thr221 residues, even if they are not optimal in all the calculated docking poses.

Since DKP-RGD compounds are highly preorganized for binding to α_V_β_6_, the modulation of their experimental affinity can be explained by considering the different orientation of the aromatic rings of the DKP scaffold within the binding site. Indeed, docking results showed that, depending on DKP scaffold (i.e., on endocyclic nitrogen N-1/N-4 substitution and carbon C-3/C-6 stereochemistry) and macrocycle conformation, the aromatic moieties differently fit into the integrin pocket.

**Figure 3:**
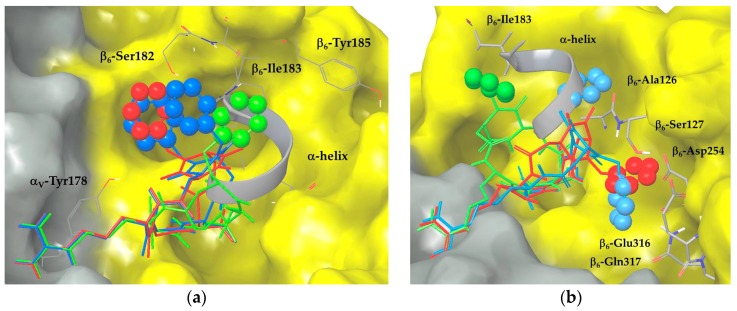
Docking best poses of (**a**) ligands **2** (red), **4** (green) and **5** (blue) and (**b**) ligands **3** (red), **6** (green) and **7** (blue) into integrin α_V_β_6_ (α_V_ surface in grey, β_6_ surface in yellow). The X-ray structure of the TGF-β3 α-helix portion is shown as a grey ribbon. Ligand aromatic rings are represented as space-filling spheres.

Docking calculations starting from type III geometry of ligand **2** place the N-4 benzyl group of the scaffold between α and β subunits interacting with the aromatic side chain of α_V_-Tyr178 and with β_6_-Ser182. This benzyl position could prevent optimal RGD interactions and perturb the whole ligand binding, thus explaining its high-nanomolar experimental binding affinity (IC_50_ = 345.0 ± 1.0 nM). A different binding mode of the aromatic moiety is observed in the docking poses of ligand **4** that displays an improved binding ability (IC_50_ = 95.3 ± 4.9 nM). The N-1 benzyl substitution and the type I macrocycle geometry generate docking poses with similar docking scores and RGD interactions with respect to ligand **2**, but with a different orientation of the benzyl group that interacts with β_6_-Ile183, a ‘hot spot’ residue of the TGF-β3 α-helix pocket. By placing the aromatic ring in the α-helix region, ligand **4** seems to be more effective in displacing the natural ligand in the competitive binding assay. In line with these considerations, in the docking poses of the di-benzylated compound **5** (IC_50_ = 173.5 ± 52.5 nM), the aromatic moieties partially fit both of the benzyl binding regions identified by ligands **2** and **4**. When compared to the mono-benzylated compounds, the addition of a second aromatic group improves the experimental binding affinity only with respect to ligand **2**, while it has a perturbing effect with respect to ligand **4**. In fact, the docking poses of ligand **5**, adopting the type III geometry, show one benzyl group placed between α_V_-Tyr178 and β_6_-Ser182 (as in ligand **2**), and the second one exposed to the solvent or in contact with β_6_-Ile183 (as in ligand **4**). The absence of a stable interaction of the benzyl moieties with key residues of the hydrophobic β_6_ pocket is probably responsible for the intermediate affinity of **5** for the receptor. The superimposition of the best poses of the three ligands (featuring the same 3R, 6S scaffold configuration) is shown in Figure 3a.

In the docking poses of ligand **3**, featuring the type III macrocycle geometry and the N-4 benzyl substitution, the aromatic moiety is shifted towards β_6_-Glu316, β_6_-Gln317, and β_6_-Asp254 residues close to the ADMIDAS (adjacent to MIDAS) ion, forming a large number of favorable contacts with the protein. When compared to ligand **4**, the benzyl group fits a different protein region that stabilizes the binding of the RGD motif, as suggested by the experimental receptor affinity (IC_50_ = 95.6 ± 24.6 nM). For ligand **6**, the switch to type I macrocycle conformation and to N-1 benzyl substitution produces ligand poses with the aromatic ring in contact with β_6_-Ile183 of the α-helix pocket. Such a binding mode corresponds to the docking pose observed for ligand **4** and nicely agrees with the experimental IC_50_ value of 76.6 ± 4.2 nM, suggesting that the position of the aromatic ring close to α-helix hydrophobic region has a stabilizing effect on ligand binding. The docking poses of ligand **7**, the most active compound of the library exhibiting a low nanomolar IC_50_ value, show some similarities with both ligands **3** and **6**. The presence of two aromatic rings improves the experimental integrin affinity as compared to the mono-benzylated analogs, because both benzyl groups productively contribute to ligand stabilization. In particular, ligand **7**, adopting the type I geometry, docks one benzyl group in the protein region explored by ligand **3**, forming contacts with β_6_-Glu316 and β_6_-Gln317 residues, and the other one in the α-helix hydrophobic pocket, nicely overlapping the α-helix region and forming contacts with β_6_-Ile183, β_6_-Ala126, and β_6_-Ser127. Interestingly, ligand **7** displays the best docking score among all of the investigated compounds (see the Supplementary Materials, Table S1) and the highest number of docking poses maintaining all the crystallographic RGD interactions. The superimposition of the best poses of the three ligands featuring the same 3S, 6R scaffold configuration is shown in Figure 3b.

Finally, docking calculations on compound **8** (c[DKP-3-RAD], used as a negative control), starting from the type III geometry, fail in reproducing the X-ray key interactions of Asp and Arg residues. In the best pose of the stereoisomer containing the (*S*)-Ala amino acid the carboxylate group coordinates the ADMIDAS Ca^2+^ ion, while the guanidinium group form a π-cation interaction with α_V_-Tyr178 side chain. In the best pose of the stereoisomer containing the (*R*)-Ala residue, the coordination of the Asp side chain to MIDAS Mg^2+^ is kept (without hydrogen bonds to β_6_ residues), while the Arg side chain interacts with α_V_-Asp148 and α_V_-Asp150. Accordingly, the docking scores of the RAD peptidomimetics are about 1–3 kcal/mol worse than those calculated for the RGD ligands (see the Supplementary Materials, Table S1).

## 3. Discussion

In view of the roles played by integrin α_V_β_6_ in cancer growth and progression [5,6,7,8,9,41], small-molecule integrin antagonists may be a valuable tool to modulate these processes. Investigating the interaction of cyclic RGD peptidomimetics with this integrin subtype represents the first essential step towards their exploitation as inhibitors of integrin functions and as receptor ligands for targeted therapy and imaging of tumors. The cyclic DKP-RGD peptidomimetics **2**–**7** have been designed to target the α_V_β_3_ integrin, qualifying as excellent ligands (from low- to sub-nanomolar IC_50_ values in the inhibition of biotinylated vitronectin binding to α_V_β_3_) of this RGD-recognizing heterodimer [24]. Prompted by the similarity of the ligand RGD binding observed in the crystal structures of Cilengitide to α_V_β_3_ and TGF-β3 peptide to α_V_β_6_ [32,37], we decided to investigate the interaction of the cyclic peptidomimetics with the α_V_β_6_ integrin by means of competitive cell-free binding assays and docking studies. Indeed, the favored geometries of these ligands are characterized by an extended arrangement of the RGD sequence comparable to the X-ray α_V_β_6_-bound RGD conformation and suitable to establish useful interactions with α_V_β_6_ integrin.

Accordingly, docking calculations of the cyclic peptidomimetics in the α_V_β_6_ crystal structure predicted RGD binding modes reproducing the key interactions found in the X-ray complex of TGF-β3 peptide to α_V_β_6_. In particular, the electrostatic clamp of ligand Arg and Asp side chains with the corresponding charged regions in the α_V_ and β_6_ receptor subunits is properly formed, in combination with a stabilizing network of hydrogen bonds whose specific features depend on the structural properties of each particular ligand (e.g., scaffold substitution and stereochemistry).

However, similar to what is observed for cyclic RGD peptides, the peptidomimetics exhibited binding affinities for the α_V_β_6_ integrin (measured as the concentration of compound required for 50% inhibition of biotinylated fibronectin binding to isolated α_V_β_6_ integrin) about 10–100 times lower than those for the related α_V_β_3_ receptor (Table 1). Although the RGD binding mode found in α_V_β_6_ integrin recapitulates the RGD interaction with α_V_β_3_, differences between integrin binding pockets can affect the ligand recognition and binding ability. For instance, β_3_-Tyr122 residue, which is engaged into a π-interaction with the D-Phe residue of Cilengitide [37] or with a suitable aromatic moiety of the cyclic DKP-RGD peptidomimetics **2**–**7** [24], is mutated into β_6_-Ala126, hampering an important contribution to the complex stabilization.

More importantly, further sequence differences between the β subunits create a β_6_-specific hydrophobic pocket that was shown to play a key role in the elucidation of specificity determinants of integrin β subunits [32]. In particular, three βI-domain loops contribute to the main difference in the ligand-binding region between α_V_β_6_ and α_V_β_3_ [32]. The amphipathic α-helix of the TGF-β3 undecapeptide makes extensive contacts with these three loops, fully exploiting the hydrophobic pocket composed only of residues from the β_6_ subunit, and acting to stabilize the RGD interaction. Interestingly, in contrast to the α_V_β_6_ complex, complexes of α_V_β_3_, α_IIb_β_3_, and α_5_β_1_ exhibit little interaction beyond that with the RGD motif [37,38,39,40].

As shown by docking results, the cyclic DKP-RGD peptidomimetics **2**–**7** can only partially take advantage of the structural peculiarity of α_V_β_6_ integrin, fitting the hydrophobic pocket to an incomplete extent thanks to the structural features of specific ligands. In particular, the analysis of the docking poses suggests that the interactions of the DKP benzyl moieties with key residues of the hydrophobic β_6_ pocket as well as their overlap with the α-helix region of the TGF-β3 peptide, correlate well with the ability of the cyclic peptidomimetics to displace the natural ligand in competitive binding assays. Indeed, ligand **7**, displaying the best fit to the TGF-β3 peptide and the most favorable interactions with α_V_β_6_ integrin for both the RGD and not-RGD portions in the calculated binding modes, appears the best α_V_β_6_ ligand of the library, inhibiting the binding of biotinylated fibronectin to α_V_β_6_ at a low nanomolar IC_50_ value (2.3 ± 0.8 nM).

Recently, a cyclic RGD peptide endowed with sub-nanomolar binding affinity toward the α_V_β_6_ integrin and a remarkable selectivity against other integrins has been reported as a result of a strategy based on the grafting of the epitope from the α_V_β_6_ binding helix onto a cyclic β-sheet structure [42]. Although, in contrast to linear peptides [43], the DLXXL-motif was not essential for the α_V_β_6_ activity of the cyclic peptides, three RGD-flanking hydrophobic residues were shown to significantly contribute to the interaction, fitting the wide hydrophobic pocket and projecting their side chains in the same direction as the key residues of the TGF-β3 helical motif [42].

In conclusion, experimental and computational tools for the evaluation of α_V_β_6_ integrin ligands have been set up and then applied to investigate the interaction of cyclic RGD peptidomimetics with α_V_β_6_ integrin. In particular, a docking protocol was defined and then exploited to predict the binding mode of the cyclopeptides to the α_V_β_6_ integrin, generating poses that fairly reflect the results of the competitive binding assays to the isolated receptor. Insights from the features of the binding pocket combined with the analysis of the docking poses provided a rationale for ligand recognition and enabled to outline the molecular bases of ligand binding affinities. This understanding might in turn be exploited to develop α_V_β_6_-selective ligands and improved targeting agents for biomedical applications.

## 4. Materials and Methods

### 4.1. Integrin Ligands

The cyclic peptidic and peptidomimetic RGD ligands used in this work were purchased or synthesized according to published procedures [17,24]. The synthesis and the characterization of the new cyclic peptidomimetic c[DKP-3-RAD] **8** are reported in the Supplementary Materials (Scheme S1, Figures S1–S6).

### 4.2. Solid-Phase Receptor Binding Assay

Recombinant human integrin α_V_β_6_ receptor (R&D Systems, Minneapolis, MN, USA) was diluted to 1.0 μg/mL in coating buffer containing 20 mM Tris-HCl (pH 7.4), 150 mM NaCl, 1 mM MnCl_2_, 2 mM CaCl_2_, and 1 mM MgCl_2_. An aliquot of diluted receptor (100 μL/well) was added to 96-well microtiter plates (Nunc MaxiSorp, Thermo Fisher Scientific, Roskilde, Denmark) and incubated overnight at 4 °C. The plates were incubated with blocking solution (coating buffer plus 1% bovine serum albumin) for additional 2 h at room temperature to block nonspecific binding. After washing twice with blocking solution, plates were incubated shaking in the dark for 3 h at room temperature, with various concentrations (10^−4^–10^−11^ M) of test compounds in the presence of 1 μg/mL biotinylated fibronectin (Molecular Innovations, Novi, MI, USA). Biotinylation was performed using an EZ-Link Sulfo-NHS-Biotinylation kit (Pierce, Rockford, IL, USA). After washing three times, the plates were incubated shaking for 1 h, at room temperature, with streptavidin-biotinylated peroxidase complex (Amersham Biosciences, Uppsala, Sweden). After washing 3 times with blocking solution, plates were incubated with 100 μL/well of Substrate Reagent Solution (R&D Systems) for 30 min shaking in the dark, before stopping the reaction with the addition of 50 μL/well 2N H_2_SO_4_. Absorbance at 415 nm was read in a SynergyTM HT Multi-Detection Microplate Reader (BioTek Instruments, Inc., Winooski, VT, USA). Each data point represents the average of triplicate wells; data analysis was carried out by nonlinear regression analysis with GraphPad Prism software (GraphPad Software, Inc., La Jolla, CA, USA). Each experiment was repeated in triplicate.

### 4.3. Computational Studies

*Protein setup.* The crystal structure of the extracellular domain of the integrin α_V_β_6_ in complex with the HGRGDLGRLKK undecapeptide of the TGF-β3 prodomain (PDB code: 4UM9) [32] was used for docking studies. Docking was performed only on the globular head of the integrin, because the headgroup of integrin has been identified in the X-ray structure as the ligand-binding region. The protein was truncated to residue sequences 1–439 for chain α (chain C of crystal asymmetric unit) and 114–355 for chain β (chain D of crystal asymmetric unit). According to the X-ray structure, the bivalent cation at MIDAS has been modeled as Mg^2+^ ion, whereas all of the other metal cations were modeled as Ca^2+^ ions. All waters molecules were deleted except for the three water molecules coordinating the MIDAS cation and the single water molecule found around ADMIDAS ion. The structure was then prepared by using the Protein Preparation Wizard of the graphical user interface Maestro and the OPLSAA force field [44]. Hydrogen bonds were optimized according to the exhaustive sampling option and the entire complex was optimized by using a restrained minimization with convergence on heavy atoms to a RMSD (root-mean-square deviation) of 0.30 Å.

*Ligand docking calculations.* The automated docking calculations were performed by using Glide V5.7 in the standard precision (SP) mode [35]. The grids were generated for the RGD-integrin α_V_β_6_ complex structure prepared as described in the protein setup section. The center of the grid-enclosing box was defined by the center of the bound ligand. For the grid generation step, the size of the inner cubic box for placing the ligand center was set to 12 Å, and a value of 26 Å was used for the outer cubic box. The outer box dimensions fit the entire active site. No further modifications were applied to the default settings. For the docking calculations, the GlideScore function was used to select 20 poses for each ligand after a post-minimization step. The flexible docking option was selected and the SP modality was used with amide bonds set to trans configurations. No Epik state penalty was added to the docking score and all of the ligands were considered in their zwitterionic form (and protonated Lys residue for **1c**). To validate the docking protocol, a known α_V_β_6_ ligand was selected, i.e., the cyclic pentapeptide c[RGDfK] **1c**, showing an IC_50_ value to the isolated receptor of 52.0 ± 23.8 nM (see Table 1). In fact, due to the high conformational flexibility, the X-ray ligand (the undecapeptide of the TGF-β3 prodomain) is not suitable for standard docking calculations. For compound c[RGDfK] **1c**, Glide succeeded in reproducing the experimentally determined binding mode of the RGD motif, as it corresponds to the best-scored pose (see Figure 2b).

*Ligand conformations.* The conformations of the ligands used in docking studies are described in the Appendix A. To avoid incomplete sampling of macrocycle conformations during docking analyses, the assessment of the preferred conformations of the cyclic systems has been performed as a separate step before docking [45].

## Figures and Tables

**Table 1:** Inhibition of biotinylated fibronectin binding to α_V_β_6_ integrin compared with inhibition of biotinylated vitronectin binding to α_V_β_3_.

Compound	α_V_β_6_ IC_50_ [nM] ^1^	α_V_β_3_ IC_50_ [nM] ^2^	IC_50_ (α_V_β_6_)/IC_50_ (α_V_β_3_)
**1a** c[RGDf(N-Me)V]	82.8 ± 4.9	0.71 ± 0.06	117
**1b** c[RGDfV]	104.7 ± 18.9	3.2 ± 1.3	33
**1c** c[RGDfK]	52.0 ± 23.8	1.4 ± 0.2	37
**2**	345.0 ± 1.0	3.2 ± 2.7	108
**3**	95.6 ± 24.6	4.5 ± 1.1	21
**4**	95.3 ± 4.9	7.6 ± 4.3	13
**5**	173.5 ± 52.5	12.6 ± 5.0	14
**6**	76.6 ± 4.2	2.1 ± 0.6	37
**7**	2.3 ± 0.8	0.2 ± 0.09	12
**8** c[DKP-3-RAD]	4095 ± 1425	1500 ± 540	3

^1^ IC_50_ values were calculated as the concentration of compound required for 50% inhibition of biotinylated fibronectin binding as estimated by GraphPad Prism software; all values are the arithmetic mean ± SD of triplicate determinations. ^2^ Calculated as the concentration of compound required for 50% inhibition of biotinylated vitronectin binding [24].

## Supplementary Materials

The following are available online at http://www.mdpi.com/2072-6694/9/10/128/s1. Scheme S1: Synthesis of compound **8**, Figures S1–S6: HPLC traces and NMR spectra of compound **8**, Figures S7–S9: Preferred conformations identified for the cyclic [DKP-RGD] peptidomimetics (2D and 3D representations), Table S1: Glide docking score values of the best poses.

## Appendix A

Docking studies were performed starting from the preferred macrocycle conformations of the cyclic DKP-RGD peptidomimetics previously determined [24,46]. Four different geometries (denoted as type I–IV) were identified in the free state conformational analysis of the cyclic RGD ligands containing the DKP scaffolds, by means of computational and spectroscopic NMR studies, as summarized in the Supplementary Materials. Depending on the configuration and substitution of the DKP scaffold, the cyclic DKP-RGD ligands showed different intramolecular H-bonding patterns as characterized by specific β- and γ-turns and diverse arrangements of the RGD sequence. In Figure A1, the type I and III patterns are shown as they correspond to the preferred macrocycle conformations adopted by the compounds under investigation. Docking calculations of ligand **6** were also run from type IV geometry, obtaining results worse than those provided by type I.

As the NMR-based solution structures of **1a** (Cilengitide) exhibit conformations closely resembling the crystal structure bound to the head group of integrin α_V_β_3_, docking calculations were performed from the X-ray α_V_β_3_-bound geometry [47]. Interestingly, the type I conformation of the cyclic DKP-RGD peptidomimetics is very similar to this geometry.

Metropolis Monte Carlo/Stochastic Dynamics (MC/SD) simulations [48], using the implicit water GB/SA solvation model [49], were performed on ligand **1c** within the framework of MacroModel V9.9 [50]. MC/SD simulations were performed at 300 K using the all-atoms AMBER* force field and a time step of 1 fs for 20 ns of simulation time. Side-chain dihedral angles were defined as internal coordinate degrees of freedom in the Monte Carlo part of the algorithm. A total of 5000 conformations were stored for analysis. To assess the convergence of the calculations, three independent MC/SD simulations were run starting from different macrocycle conformations. The analysis of the sampled structures showed that during all of the simulations compound **1c** preferentially adopts an extended arrangement of the RGD sequence, characterized by a β-turn centered on Gly-Asp residues, comparable to the X-ray α_V_β_3_–bound geometry of Cilengitide. Macrocycle conformations featuring a β-turn at D-Phe-Lys or a β-turn at Lys-Arg were also sampled during the MC/SD simulations. Three representative macrocycle conformations were then used for docking calculations and the best results were obtained starting from the preferred Gly-Asp β-turn conformation.
