# Supplementary material for: Investigating the Interaction of Cyclic RGD Peptidomimetics with αVβ6 Integrin by Biochemical and Molecular Docking Studies
Source: Cancers (Basel). 2017 Sep 21;9(10):128. doi: 10.3390/cancers9100128 (PMC5664067; doi:10.3390/cancers9100128)
Supplement: Supplementary file 1 [file cancers-09-00128-s001.docx]

Supplementary Materials: Investigating the Interaction of Cyclic RGD Peptidomimetics with α_V_β_6_ Integrin by Biochemical and Molecular
Docking Studies

Monica Civera, Daniela Arosio, Francesca Bonato, Leonardo Manzoni, Luca Pignataro,
Simone Zanella, Cesare Gennari, Umberto Piarulli and Laura Belvisi

**Synthesis of cyclo[DKP-3-RAD] 8A and 8B**

The solution-phase synthesis of **8** is shown in Scheme S1. N_3_-DKP3-COOAllyl **13**, BocHN-DKP3-COOAllyl **14** and BocHN-DKP3-COOH **15** were synthesized according to literature procedure and their analytical data were in agreement with those already published [23,24].

**Scheme S1.** Reagents and conditions. (a) HATU, HOAt, *i*Pr_2_NEt, DMF, 0 °C to r.t., overnight, 84%; (b) TFA, CH_2_Cl_2_, r.t., 2 h; (c) PMe_3_ in toluene, Boc-ON, THF, -20 °C to r.t., 5 h, 78%; (d) [Pd(PPh_3_)_4_], pyrrolidine, PPh_3_, CH_2_Cl_2_, 0 °C, 1 h, quant.; (e) **15**, HATU, HOAt, *i*Pr_2_NEt, DMF, 0 °C to r.t., overnight, 65%; (f) TFA, CH_2_Cl_2_, r.t., 2 h; (g) Cbz-l-Asp(O*t*Bu)-OH, HATU, HOAt, *i*Pr_2_NEt, DMF, 0 °C to r.t., overnight, 83%; (h) H_2_, 10% Pd/C, THF/H_2_O 1:1, overnight, r.t., quant.; (i) HATU, HOAt, *i*Pr_2_NEt, DMF/CH_2_Cl_2_ 1:1 (1.4 mm), 0 °C to r.t., overnight, 82%; (j) TFA/thioanisole/EDT/anisole 90:5:3:2, 2 h, r.t., 30%. The synthesis was performed according to the procedure used for the correponding RGD cyclopeptide [23,24].

BocHN-Arg(Mtr)-Ala-OBn (**11**)

To a solution of BocHN-Arg(Mtr)-OH **9** (290 mg, 0.60 mmol, 1.0 eq) in DMF (9.0 mL), at 0 °C and under nitrogen atmosphere, HATU (250 mg, 0.66 mmol, 1.1 eq), HOAt (90 mg, 0.66 mmol, 1.1 eq) and *i*Pr_2_NEt (315 µL, 1.8 mmol, 3.0 eq) were added: the reaction was stirred at 0 °C for 30 min. A solution of l-Ala-OBn∙HCl (140 mg, 0.66 mmol, 1.1 eq) in DMF (3.0 mL) and *i*Pr_2_NEt (209 µL, 1.2 mmol, 2.0 eq) was added dropwise to the previous solution: the reaction mixture was stirred at 0 °C for 1 h and at room temperature overnight. The mixture was diluted with AcOEt (50 mL) and washed with KHSO_4_ 1 M (4 × 50 mL), saturated aqueous NaHCO_3_ (4 × 50 mL) and brine (2 × 50 mL). The organic phase was dried over Na_2_SO_4_ and volatiles were removed under reduced pressure. The residue was purified by flash chromatography on silica gel (Hexane/AcOEt 3:7, solid load) affording the desired product **11** as a white foam (332 mg, 84%).

*R*_f_ = 0.13 (Hexane/AcOEt 3:7); ^1^H-NMR (400 MHz, CDCl_3_) δ 7.41 (d, J = 6.9 Hz, 1H), 7.37 – 7.26 (m, 5H), 6.52 (s, 1H), 6.31 (bs, 2H), 5.52 (d, J = 7.5 Hz, 1H), 5.12 (d, J = 12.4 Hz, 2H), 4.61 – 4.46 (m, 1H), 4.27 (bs, 1H), 3.81 (s, 3H), 3.19 (bs, 2H), 2.68 (s, 3H), 2.61 (s, 3H), 2.12 (s, 4H), 1.88 – 1.76 (m, 1H), 1.67 – 1.52 (m, 3H), 1.40 (s, 12H). ^13^C-NMR (101 MHz, CDCl_3_) δ 173.0, 172.4, 158.7, 156.4, 156.1, 138.8, 136.9, 135.5, 128.7, 128.5, 128.2, 125.0, 111.9, 80.1, 67.3, 55.6, 53.4, 48.5, 40.7, 30.6, 28.5, 25.2, 24.3, 18.5, 17.4, 12.1. MS (ESI) m/z calcd. for [C_31_H_45_N_5_NaO_8_S]^+^: 670.29; found: 670.09 [M+Na]^+^.

BocHN-DKP3-Arg(Mtr)-Ala-OBn (**16**)

To a solution of BocHN-DKP3-COOH **15** (160 mg, 0.41 mmol, 1.0 eq), prepared as described in [24], in DMF (4.1 mL), under nitrogen atmosphere and at 0 °C, HATU (186 mg, 0.49 mmol, 1.2 eq), HOAt (67 mg, 0.49 mmol, 1.2 eq) and *i*Pr_2_NEt (271 μL, 1.64 mmol, 4.0 eq.) were added successively. After 30 min, a solution of the TFA salt **12** (324 mg, 0.49 mmol, 1.2 eq), obtained treating **11** according to GP1, in DMF (4.1 mL) was added and the reaction mixture was stirred at 0 °C for 1 h and at r.t. overnight. The mixture was afterwards diluted with AcOEt (50 mL) and washed with KHSO_4_ 1 M (3 × 90 mL), saturated aqueous NaHCO_3_ (3 × 90 mL) and brine (2 × 90 mL). The organic phase was dried over Na_2_SO_4_ and volatiles were removed under reduced pressure. The residue was purified by flash chromatography on silica gel (from CH_2_Cl_2_/MeOH 96:4 to CH_2_Cl_2_/MeOH 9:1, solid load) affording the desired product **16** as a white foam (246 mg, 65%).

*R*_f_ = 0.29 (CH_2_Cl_2_/MeOH 95:5); ^1^H-NMR (400 MHz, CD_2_Cl_2_) δ 7.92 (bs, 1H), 7.79 – 7.41 (m, 2H), 7.37 – 7.15 (m, 10H), 6.54 (s, 1H), 6.25 (bs, 2H), 5.65 (bs, 1H), 5.43 – 5.34 (m, 1H), 5.09 (q, J = 12.3 Hz, 2H), 4.60 (bs, 1H), 4.54 – 4.34 (m, 2H), 4.08 (d, J = 15.0 Hz, 1H), 3.92 – 3.68 (m, 3H), 3.70 – 3.36 (m, 2H), 3.24 – 2.78 (m, 4H), 2.64 (s, 3H), 2.57 (s, 3H), 2.09 (s, 3H), 1.78 (bs, 1H), 1.60 (bs, 1H), 1.56 – 1.45 (m, 2H), 1.36 (s, 12H). ^13^C-NMR (101 MHz, CD_2_Cl_2_) δ 173.0, 172.4, 170.5, 168.1, 166.8, 158.9, 157.0, 156.5, 138.9, 136.9, 136.2, 136.0, 133.9, 129.2, 128.9, 128.7, 128.4, 125.2, 112.1, 80.2, 67.4, 60.3, 55.8, 53.0, 51.8, 48.9, 47.7, 41.2, 40.9, 38.3, 30.3, 28.5, 25.4, 24.3, 18.6, 17.4, 12.1. MS (ESI) m/z calcd. for [C_45_H_60_N_8_NaO_11_S]^+^: 943.40; found: 943.61 [M+Na]^+^.

CbzHN-Asp(OtBu)-DKP3-Arg(Mtr)-Ala-OBn (**18**)

To a solution of Cbz-l-Asp(OtBu)-OH (136 mg, 0.42 mmol, 1.5 eq) in DMF (2.6 mL), under nitrogen atmosphere and at 0 °C, HATU (160 mg, 0.42 mmol, 1.5 eq), HOAt (57 mg, 0.42 mmol, 1.5 eq) and *i*Pr_2_NEt (195 μL, 1.12 mmol, 4.0 eq) were added successively. Compound **16** (255 mg, 0.28 mmol, 1.0 eq) was deprotected according to GP1 and the corresponding trifluoroacetate salt **17** was dissolved in DMF (3.0 mL) and added to the previous solution after 30 min. The reaction mixture was stirred at 0 °C for 1 h and at r.t. overnight. The mixture was afterwards diluted with AcOEt (45 mL) and washed with KHSO_4_ 1 M (3 × 40 mL), saturated aqueous NaHCO_3_ (3 × 40 mL) and brine (1 × 40 mL). The organic phase was dried over Na_2_SO_4_ and volatiles were removed under reduced pressure. The residue was purified by flash chromatography on silica gel (CH_2_Cl_2_/MeOH 95:5, solid load) affording the desired product **18** as a white foam (263 mg, 83%).

*R*_f_ = 0.27 (CH_2_Cl_2_/MeOH 95:5); ^1^H-NMR (400 MHz, CD_2_Cl_2_) δ 7.72 (bs, 1H), 7.59 (bs, 2H), 7.39 – 7.16 (m, 15H), 6.53 (s, 1H), 6.25 (bs, 2H), 6.17 (d, J = 7.5 Hz, 1H), 5.23 (d, J = 15.2 Hz, 1H), 5.16 – 5.01 (m, 3H), 4.88 (d, J = 12.0 Hz, 1H), 4.63 – 4.51 (m, 2H), 4.50 – 4.39 (m, 3H), 4.15 (d, J = 15.2 Hz, 1H), 3.86 – 3.81 (m, 2H), 3.79 (s, 3H), 3.63 – 3.51 (m, 1H), 3.17 – 3.04 (m, 2H), 2.99 (d, J = 14.0 Hz, 1H), 2.81 (d, J = 14.0 Hz, 1H), 2.77 – 2.68 (m, 1H), 2.64 (s, 3H), 2.57 (s, 3H), 2.09 (s, 3H), 1.86 – 1.74 (m, 1H), 1.67 – 1.56 (m, 1H), 1.51 (s, 2H), 1.34 (s, 12H). ^13^C-NMR (101 MHz, CD_2_Cl_2_) δ 173.1, 172.3, 172.1, 170.9, 170.7, 168.0, 166.8, 158.8, 156.9, 156.8, 138.8, 136.9, 136.6, 136.3, 136.0, 134.0, 129.2, 128.9, 128.7, 128.5, 128.4, 128.2, 125.1, 112.1, 82.0, 67.6, 67.4, 59.9, 55.8, 53.0, 52.1, 51.7, 48.9, 47.9, 40.9, 40.0, 38.5, 37.5, 30.3, 30.1, 28.1, 25.3, 24.3, 18.6, 17.4, 12.1. MS (ESI) m/z calcd. for [C_56_H_71_N_9_NaO_14_S]^+^: 1148.47; found: 1148.58 [M+Na]^+^.

CbzHN-Asp(OtBu)-DKP3-Arg(Mtr)-Ala-OBn (**19**)

Cbz-carbamate and benzyl ester protecting groups were removed by hydrogenolysis of compound **18** as described in GP2. The desired product was obtained as white foam (185 mg, quantitative). MS (ESI) m/z calcd. for [C_41_H_59_N_9_NaO_12_S]^+^: 924.39; found: 924.61 [M+Na]^+^.

Cyclo[DKP3-Arg(Mtr)-Ala-Asp(OtBu)] (**20A** + **20B**)

The macrolactamization reaction on **19** was performed according to GP3. The crude compound was dissolved in AcOEt (150 mL) and washed with KHSO_4_ 1 M (3 × 100 mL). The organic layer was dried over Na_2_SO_4_ and volatiles were removed under reduced pressure. The residue was purified by flash chromatography on silica gel (from CH_2_Cl_2_/MeOH 96:4 to CH_2_Cl_2_/MeOH 92:8, solid load) affording the desired product as a mixture of two inseparable isomers **20A** and **20B** as a white foam (150 mg, 82%). We assume that epimerization at the alanine stereocenter occurred during the amide bond formation.

*R*_f_ = 0.60, 0.52 (CH_2_Cl_2_/MeOH 9:1); ^1^H-NMR (400 MHz, CD3OD) δ 7.39 – 7.21 (m, 5HA + 5HB), 6.66 (s, 1HA), 6.65 (s, 1HB), 5.51 (d, J = 17.8 Hz, 1HA), 5.29 – 5.12 (m, 1HB), 4.74 (d, J = 6.4 Hz, 1HB), 4.60 (bs, 2HA), 4.58 – 4.49 (m, 1HB), 4.45 – 4.34 (m, 1HA), 4.20 – 4.03 (m, 1HA + 3HB), 4.03 – 3.89 (m, 2HA + 1HB), 3.89 – 3.79 (m, 4HA + 4HB), 3.64 (bs, 1HA), 3.61 – 3.50 (m, 1HB), 3.41 (dd, J = 14.0, 5.0 Hz, 1HB), 3.28 – 3.12 (m, 1HA + 2HB), 3.12 – 3.03 (m, 2HA), 3.02 – 2.70 (m, 3HA+ 2HB), 2.68 (s, 3HA), 2.67 (s, 3HB), 2.61 (s, 3HA), 2.60 (s, 3HB), 2.52 – 2.38 (m, 1HB), 2.36 – 2.20 (m, 1HB), 2.13 (s, 3HA), 2.10 (s, 3HB), 1.95 – 1.75 (m, 1HA + 2HB), 1.75 – 1.52 (m, 3HA + 2HB), 1.52 – 1.36 (m, 12HA + 12HB). ^13^C-NMR (101 MHz, CD_3_OD) δ 175.0, 174.1, 173.5, 172.3, 171.5, 170.7, 169.4, 168.6, 159.9, 158.1, 139.5, 137.9, 137.3, 136.6, 134.9, 130.7, 130.0, 129.9, 129.4, 129.1, 128.9, 125.7, 113.2, 112.8, 82.7, 82.5, 60.5, 60.2, 56.0, 53.2, 53.0, 47.8, 41.4, 40.5, 40.2, 39.6, 38.6, 37.1, 36.7, 30.7, 28.4, 28.3, 27.8, 24.4, 18.8, 18.2, 16.8, 12.2. MS (ESI) m/z calcd. for [C_41_H_57_N_9_NaO_11_S]^+^: 906.38; found: 906.56 [M+Na]^+^.

Cyclo[DKP-3-RAD] Peptidomimetics **8A** and **8B**

The final deprotection on the mixture **20A** + **20B** was carried out as described in GP4. The residue was purified by RP-HPLC (gradient: from 90% H_2_O + 0.1% TFA / 10% CH_3_CN + 0.1% TFA to 72% H_2_O + 0.1% TFA / 28% CH_3_CN + 0.1% TFA in 12 min, t_R_ (product **8A**) = 9.2 min, t_R_ (product **8B**) = 11.0 min). The purified products were freeze-dried from water to give **8A** (6.5 mg) and **8B** (5.7 mg) trifluoroacetate salts as a white solids (30%).

Compound **8A**: ^1^H-NMR (400 MHz, D_2_O) δ 7.56 – 7.35 (m, 5H), 5.14 (d, J = 15.7 Hz, 1H), 4.80 – 4.72 (m, 1H, overlapped with solvent signal), 4.65 (t, J = 6.8 Hz, 1H), 4.38 (q, J = 7.1 Hz, 2H), 4.25 – 4.14 (m, 3H), 3.54 (dd, J = 14.9, 4.8 Hz, 1H), 3.30 (t, J = 6.8 Hz, 2H), 3.10 (dd, J = 14.2, 5.7 Hz, 1H), 2.99 (d, J = 6.9 Hz, 2H), 2.70 (dd, J = 14.2, 6.0 Hz, 1H), 1.94 (dd, J = 14.9, 6.9 Hz, 2H), 1.85 – 1.63 (m, 2H), 1.50 (d, J = 7.2 Hz, 3H). ^13^C-NMR (101 MHz, D_2_O) δ 174.4, 173.6, 173.2, 172.8, 169.3, 168.0, 156.8, 135.3, 129.1, 128.2, 127.6, 60.3, 55.7, 52.3, 51.0, 49.9, 48.1, 40.5, 39.5, 38.2, 34.7, 26.6, 24.6, 16.0. MS (ESI) m/z calcd. for [C_27_H_38_N_9_O_8_]^+^: 616.28; found: 616.30 [M+H]^+^.

Compound **8B**: ^1^H-NMR (400 MHz, D_2_O) δ 7.51 – 7.30 (m, 5H), 5.39 (d, J = 15.6 Hz, 1H), 4.61 (t, J = 4.2 Hz, 1H), 4.54 (t, J = 6.1 Hz, 1H), 4.49 (dd, J = 8.5, 4.5 Hz, 1H), 4.17 (d, J = 15.6 Hz, 1H), 4.12 – 4.02 (m, 2H), 4.02 – 3.74 (m, 2H), 3.17 (dd, J = 15.3, 4.2 Hz, 1H), 3.09 (dt, J = 11.2, 5.5 Hz, 2H), 3.04 – 2.90 (m, 2H), 2.84 (dd, J = 15.3, 4.9 Hz, 1H), 1.93 – 1.60 (m, 2H), 1.62 – 1.46 (m, 2H), 1.41 (d, J = 7.2 Hz, 3H). ^13^C-NMR (101 MHz, D_2_O) δ 175.0, 174.4, 173.9, 173.1, 170.7, 168.0, 167.4, 156.6, 135.0, 129.2, 129.0, 128.1, 127.5, 59.3, 52.4, 51.6, 51.1, 50.8, 47.3, 40.7, 39.6, 37.6, 34.5, 29.9, 24.5, 15.9. MS (ESI) m/z calcd. for [C_27_H_38_N_9_O_8_]^+^: 616.28; found: 616.32 [M+H]^+^.

The two isomers **8A** and **8B** (obtained in *ca*. 1:1 ratio) were examined in vitro for their ability to compete with biotinylated vitronectin for binding to the isolated α_V_β_3_ integrin, showing 360 ± 40 nM and 1500 ± 540 nM IC_50_ values, respectively. The isomer **8B** exhibiting the worst micromolar IC_50_ value, was selected as a negative control for the competive binding assays to α_V_β_6_ integrin.

GP1. GENERAL PROCEDURE 1 FOR BOC DEPROTECTION REACTIONS

To a solution of the N-Boc-protected amino acid or peptide in CH_2_Cl_2_ (0.13 M) was added half volume of TFA and the reaction was stirred at r.t. for 2 h. The solvent was evaporated, toluene (two times) was added followed by evaporation, and then ether was added and evaporated to afford the corresponding TFA salt.

GP2. GENERAL PROCEDURE 2 FOR CBZ-CARBAMATE AND BENZYL ESTER HYDROGENOLYTIC CLEAVAGE

Protected compound (1.0 eq) was dissolved in a mixture of THF/H_2_O (1:1) and Pd/C 10% (0.1 eq) was added. The reaction mixture was subjected to three vacuum/hydrogen cycles and then left stirring overnight at room temperature under 1 bar of hydrogen. The mixture was filtered through Celite, and the cake was washed thoroughly with THF/H_2_O (1:1) or MeOH. The filtrate was concentrated and dried.

GP3. GENERAL PROCEDURE 3 FOR MACROLACTAMIZATION REACTIONS

HATU (4.0 eq), HOAt (4.0 eq) and *i*Pr_2_NEt (6.0 eq) were added successively to a solution of deprotected linear compound (1.0 eq) in DMF (1.4 mM), under a nitrogen atmosphere at 0 °C. After stirring the reaction mixture at 0 °C for 1 h, it was allowed to reach room temperature and stirred overnight. DMF was then removed under reduced pressure.

GP4. GENERAL PROCEDURE 4 FOR MTR AND OTBU ESTER REMOVAL

Protected macrolactam was treated for 2 h with TFA (0.01 M), in the presence of ion scavengers: thioanisole (5%), ethanedithiol (3%), anisole (2%). After TFA removal under reduced pressure, the residue was dissolved in a 1:1 mixture of diisopropyl ether/water. Phases were separated and the aqueous layer was washed several times with diisopropyl ether. The aqueous phase was concentrated under reduced pressure to give the crude product, which was purified by HPLC.

**HPLC traces and NMR spectra of cyclo[DKP-3-RAD] 8A and 8B**


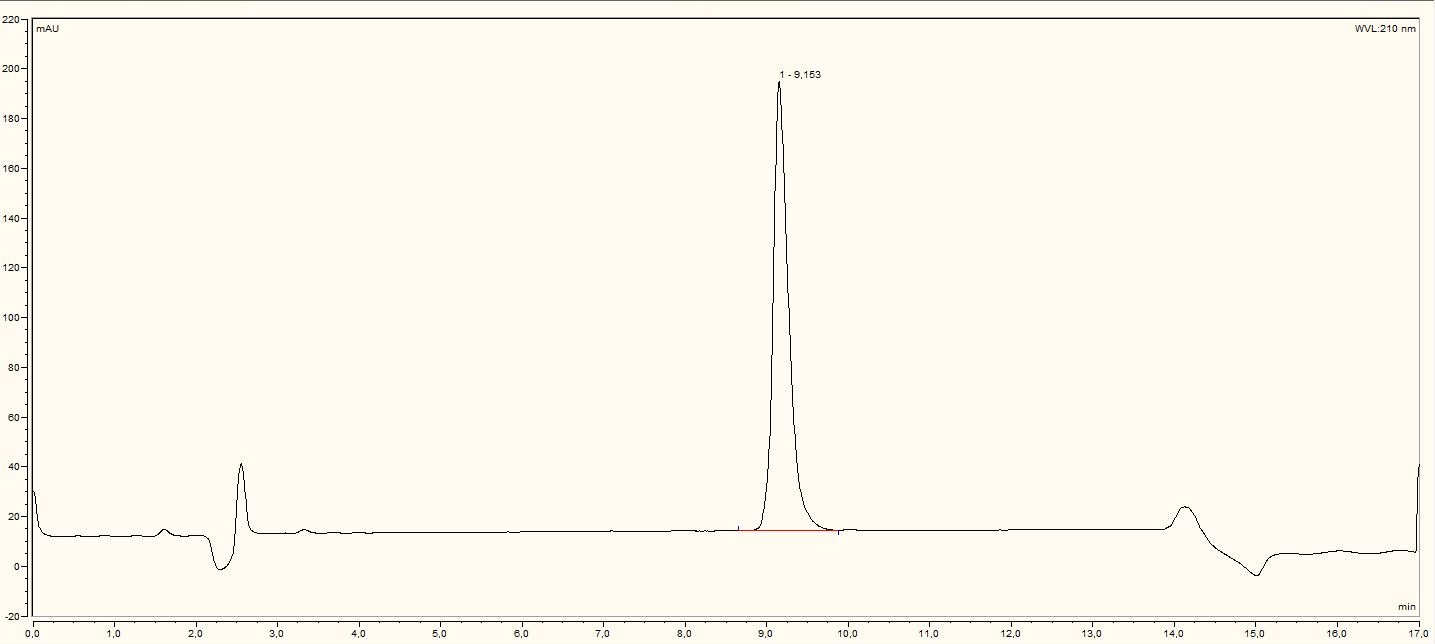


**Figure S1.** HPLC trace of cyclo[DKP-3-RAD] isomer A (**8A**) (purity: >99%).

**
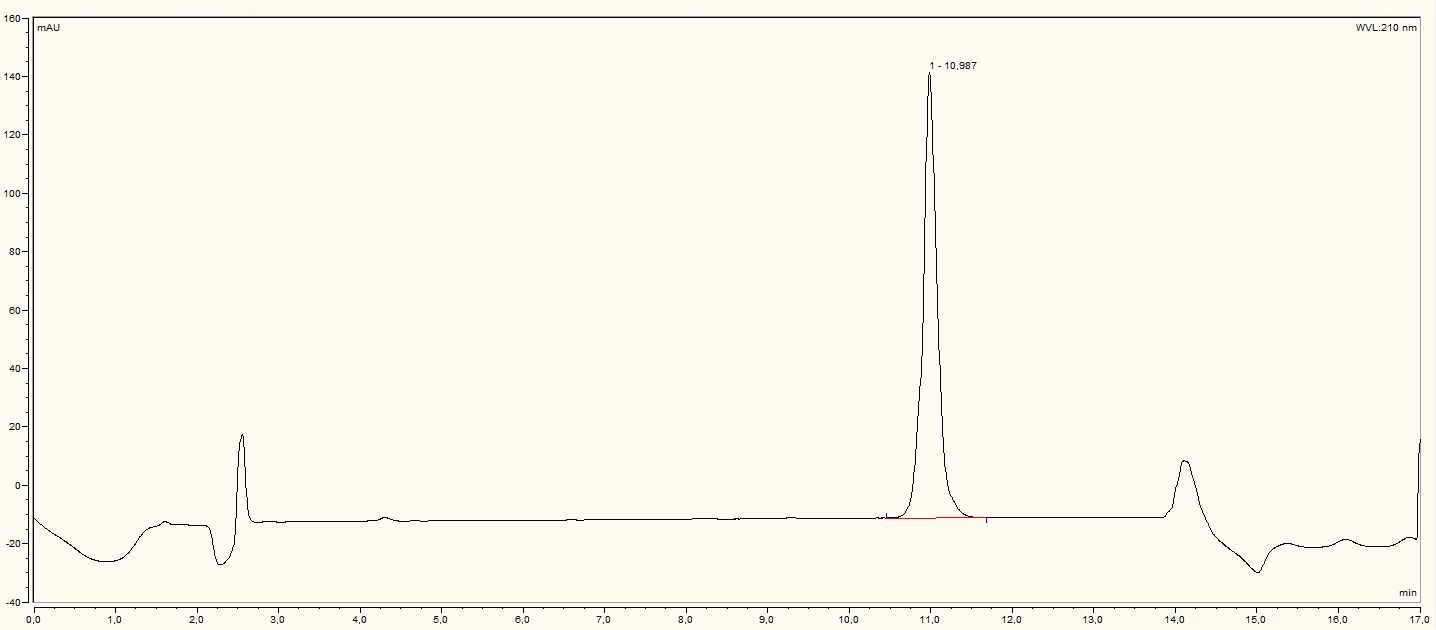
**

**Figure S2.** HPLC trace of cyclo[DKP-3-RAD] isomer B (**8B**) (purity: >99%).

**Figure S3.** ^1^H-NMR (400 MHz, D_2_O) of cyclo[DKP-3-RAD] isomer A (**8A**).

**Figure S4.** ^13^C-NMR (101 MHz, D_2_O) of cyclo[DKP-3-RAD] isomer A (**8A**).

**Figure S5.** ^1^H-NMR (400 MHz, D_2_O) of cyclo[DKP-3-RAD] isomer B (**8B**).

**Figure S6.** ^13^C-NMR (101 MHz, D_2_O) of cyclo[DKP-3-RAD] isomer B (**8B**).

**Conformational analysis of cyclo[DKP-RGD]**

The conformational studies of the cyclic RGD peptidomimetics containing the DKP scaffolds have been previously performed by means of ^1^H NMR spectroscopy experiments in phosphate buffer solution and Monte Carlo/Stochastic Dynamics (MC/SD) simulations restrained by long-range NOE contacts (OPLS_2001 force field, implicit water GB/SA solvation model) [24]. On the basis of these studies, four preferred intramolecular H-bonding patterns have been identified and denoted as type I – IV (Figure S7). Each H-bonding pattern is characterized by a β-turn (vide infra), but in addition the formation of specific γ-turns is observed during the simulations.

**Figure S7**. Preferred intramolecular hydrogen-bonded patterns identified for the cyclic [DKP-RGD] peptidomimetics on the basis of NMR and computational studies. The arrows indicate significant NOE contacts and the dotted lines the intramolecular hydrogen bonds. A) Type I H-bonding pattern, β-turn at Gly-Asp. B) Type II H-bonding pattern, β-turn at Arg-Gly. C) Type III H-bonding pattern, pseudo-β-turn at DKP-Arg. D) Type IV H-bonding pattern, pseudo-β-turn at Asp-DKP.

The type I H-bonding pattern is characterized by a β-turn motif at Gly-Asp stabilized by a hydrogen bond between DKP-NH10 and Arg-C=O and, generally, the NH10-NHAsp NOE contact is observed. The type II H-bonding pattern is described by a β-turn motif at Arg-Gly stabilized by a hydrogen bond between Asp-NH and C(8)=O and, typically, characterized by the NHAsp-NHGly NOE contact. The type III H-bonding pattern is defined by a pseudo-β-turn motif at DKP-Arg, stabilized by a hydrogen bond between NHGly and C(5)=O and the NHGly-NHArg NOE contact is detectable. Finally, the type IV H-bonding pattern is characterized by a pseudo-β-turn motif at Asp-DKP, stabilized by a hydrogen bond between NH4 and Gly-C=O (NOE contact between NH4 and NH10).

Conformational studies of compounds **2-7** revealed that the ligands display well-defined preferred conformations characterized by intramolecular hydrogen-bonded turn motifs and an extended arrangement of the RGD sequence [Cβ(Arg)-Cβ(Asp) average distances of about 9 Å]. In particular, compounds **2**, **3** and **5** preferentially adopted the type III conformation characterized by the pseudo-β-turn at DKP-Arg and the formation of the corresponding hydrogen bond between the NHGly and C(5)=O. In fact, the NOESY spectra of these ligands showed only one relevant long-range contact between NHGly and NHArg. The distance restraint corresponding to this NOE contact was applied in the MC/SD simulations and most of the conformations sampled during these simulations adopted an extended arrangement of the RGD sequence characterized by the pseudo-β-turn at DKP-Arg and the formation of the corresponding hydrogen bond between the NHGly and C(5)=O. In addition, the formation of a γ-turn at Asp stabilized by the hydrogen bond between NH10 and Gly-C=O is observed during the simulations. A representative energy minimized conformation selected by cluster analysis and featuring the type III H-bonding pattern is shown in Figure S8 for the RGD peptidomimetic **3**.


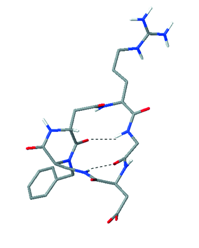


**Figure S8.** Type III conformation of compound **3** as obtained by restrained MC/SD simulations based on experimental distance information, after energy minimization (inverse γ-turn at Asp and pseudo-β-turn at DKP-Arg, Cβ(Arg)-Cβ(Asp) = 8.8 Å).

Conversely, compounds **4, 6** and **7** preferentially adopted the type I conformation characterized by the β-turn at Gly-Asp and the formation of the corresponding hydrogen bond between DKP-NH10 and Arg-C=O (and, generally, by the NH10-NHAsp NOE contact). The contribution of a second conformation (the type IV characterized by the pseudo-β-turn at Asp-DKP and the hydrogen bond between DKP-NH_4_ and Gly-C=O, see Figure S7) to the free state conformational equilibrium of ligand **6** was suggested by NMR spectroscopy data (moderate NOE contact involving NH4 and NH10 observed in the NOESY spectrum). Accordingly, three-dimensional structures satisfying long-range NOE contacts were generated for ligand **6** by performing two restrained MC/SD simulations and applying the DKP-NH10/NHAsp or the NH4/NH10 distance restraint derived from NOESY spectra. Most of the conformations sampled during the first simulation adopted an extended arrangement of the RGD sequence and are characterized by the β-turn at Gly-Asp and the corresponding hydrogen bond between DKP-NH10 and Arg-C=O. In addition, the formation of a γ-turn at Asp stabilized by the hydrogen bond between NH10 and Gly-C=O is observed during the simulation. A representative energy minimized conformation selected by cluster analysis and featuring these H bond is shown in Figure S9-A (type I H bonding pattern). Most of the conformations sampled during the simulation of **6** applying the NH4/NH10 distance restraint adopted an extended arrangement of the RGD sequence and are characterized by the pseudo-β-turn at Asp-DKP and the corresponding hydrogen bond between NH4 and Gly-C=O. In addition, the formation of a γ-turn at Asp stabilized by the hydrogen bond between NH10 and Gly-C=O is observed during the simulation. A representative energy minimized conformation selected by cluster analysis and featuring these H bonds is shown in Figure S9-B (type IV H-bonding pattern).


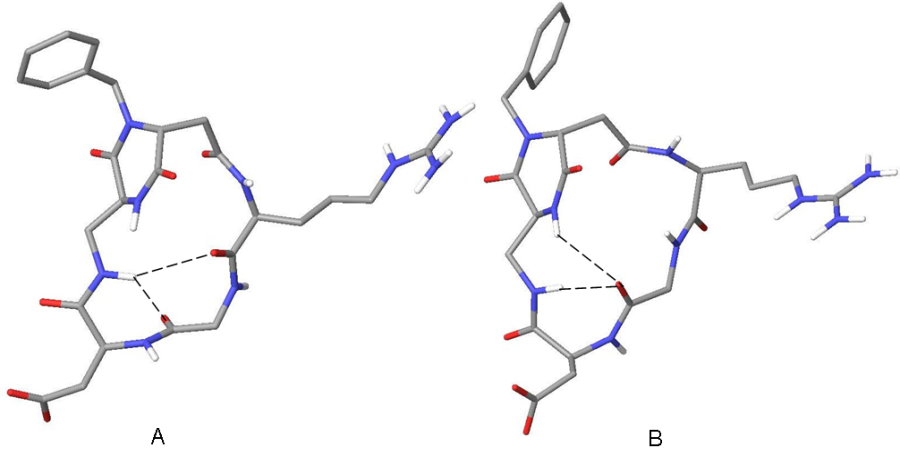


**Figure S9.** Structures of **6** as obtained by restrained MC/SD simulations based on experimental distance information, after energy minimization. A) Type I conformation (inverse γ-turn at Asp and distorted βII’-turn at Gly-Asp, Cβ(Arg)-Cβ(Asp) = 9.0 Å). B) Type IV conformation (inverse γ-turn at Asp and pseudo- β-turn at Asp-DKP, Cβ(Arg)-Cβ(Asp) = 8.8 Å).

**Glide docking score values**

**Table S1.** Glide docking score values of the best poses.

| **Compound** | **Glide docking score (kcal/mol)** |
| --- | --- |
| **1a**  c[RGDf(N-Me)V] | −9.32 |
| **1c**  c[RGDfK] | −9.01 |
| **2** | −9.23 |
| **3** | −9.22 |
| **4** | −9.62 |
| **5** | −9.91 |
| **6** | −9.32 |
| **7** | −10.25 |
| **8**  c[DKP-3-RAD] | −6.08 (*S*)-Ala; −7.89 (*R*)-Ala |

© 2017 by the authors. Licensee MDPI, Basel, Switzerland. This article is an open access article distributed under the terms and conditions of the Creative Commons Attribution (CC BY) license (http://creativecommons.org/licenses/by/4.0/).
